# Ninth Annual Commencement of the American Veterinary College

**Published:** 1884-04

**Authors:** 


NINTH ANNUAL COMMENCEMENT OF THE AMERICAN VETERINARY COLLEGE.—On Tuesday evening, March 4, the friends of the faculty and students of the American Veterinary College assembled at Chickering Hall to witness the ceremonies incident to the above interesting event.

The opening prayer having been said by the Rev. J. M. Pullman, D. D., Mr. Samuel March, President of the Board of Trustees, conferred the degrees on the gentlemen whose names are given below. The prizes were awarded by Prof. A. W. Stein, after which the Rev. John P. Newman, D. D., delivered the address.

The following are the graduates: Francis Sherwin Allen, B. S.; Armin Ernest Brum, D.V. S.; Arthur Decalb Galbraith, Elwood G. Gilbert, Wm. Henry Gribble, D. V. S.; John Hamlin, D. V. S.; Arthur Hudson Helme, Walter George Hollingworth, Isaac Newton Krowl, Morton Edward Knowles, Eldon Leon Loblein, Martin John Otto, Matthew Alexander Pierce, Edward Canfield Ross, John Elmer Ryder, Orrin William Snyder, Thomas William Spranklin, Rich'd Augustus Stoute, D. V. S.; Nicholas Pierce Valerius, Andrew Goodyear Vogt, Hamilton Vreeland, Thomas Elder White.

THE MICHIGAN STATE VETERINARY MEDICAL ASSOCIATION held its second annual meeting in the parlors of the Everett House, East Saginaw, on Tuesday, February 3d. Among those present were Drs. Hawkins, Detroit; Brenton, Jackson; McBeth, Battle Creek; Bertram, Paw Paw; Furgeson, Bay City; Grange, Lansing; Sutherland, East Saginaw; Chandler, Detroit, and Dell, Ann Arbor.

After organizing they proceeded to the election of officers, which resulted as follows: President, D. G. Sutherland, East Saginaw; First Vice-President, A. J. Murray, Detroit; Second Vice-President, J. W. Furgeson, Bay City; Third Vice-President, E. W. Bertram, Paw Paw; Rec'g Sec'y, A. J. Chandler, Detroit; Cor. Sec'y, J. A. Dell, Ann Arbor. Censors—J. Hawkins, Detroit; A. J. Murray, Detroit; C. W. Stowe, Detroit. Drs. Burt, of Flint, and Paquer, of Jackson, were received as members, while the proposals of O. L. Tick of Lapeer and James Taylor of Vassar were laid over to next meeting.

Letters were read from Drs. Murray and Jennings, Detroit, regretting their inability to be present.

After all bills were paid, leaving a balance in the treasury, $25.00 were voted to assist the passage of the bill now before Congress to prevent the spread of contagious diseases of the domestic animals.

Dr. Hawkins informed the members that he had been censured for not appointing delegates to the national convention at Chicago. As the by-laws did not give him (as President) such authority, a resolution was passed exonerating him from blame.

After much discussion, the *U. S. Vet. Journal* was retained as the organ of the association for the ensuing year. Also, that no delegates should be appointed to either the National or United States Convention until after the next meeting.

A paper on Tuberculosis and its contagious influence, was read by J. A. Dell, V. S., of Ann Arbor, which was followed by a lengthy discussion.

The usual votes of thanks were tendered the retiring officers. The Association adjourned to meet on Wednesday of the next State fair week at the same time and place.

